# Substrate-Dependent *Trans*-Stimulation of Organic Cation Transporter 2 Activity

**DOI:** 10.3390/ijms222312926

**Published:** 2021-11-29

**Authors:** Charles R. Lefèvre, Marc Le Vée, Sophie Gaubert, Elodie Jouan, Arnaud Bruyere, Caroline Moreau, Olivier Fardel

**Affiliations:** 1 CHU Rennes, Inserm, EHESP, Irset (Institut de Recherche en Santé, Environnement et Travail)-UMR_S 1085, University Rennes, 35043 Rennes, France; charles.lefevre@chu-rennes.fr (C.R.L.); caroline.moreau@chu-rennes.fr (C.M.); 2 Inserm, EHESP, Irset (Institut de Recherche en Santé, Environnement et Travail)-UMR_S 1085, University Rennes, 35043 Rennes, France; marc.levee@univ-rennes1.fr (M.L.V.); sophie.gaubert@etudiant.univ-rennes1.fr (S.G.); elodie.jouan@univ-rennes1.fr (E.J.); arnaud.bruyere@univ-rennes1.fr (A.B.)

**Keywords:** solute carrier, transporter, substrate, *trans*-stimulation, *cis*-inhibition, OCT2, DiASP

## Abstract

The search of substrates for solute carriers (SLCs) constitutes a major issue, owing notably to the role played by some SLCs, such as the renal electrogenic organic cation transporter (OCT) 2 (*SLC22A2*), in pharmacokinetics, drug–drug interactions and drug toxicity. For this purpose, substrates have been proposed to be identified by their *cis*-inhibition and *trans*-stimulation properties towards transporter activity. To get insights on the sensitivity of this approach for identifying SLC substrates, 15 various exogenous and endogenous OCT2 substrates were analysed in the present study, using 4-(4-(dimethylamino)styryl)-N-methylpyridinium iodide (DiASP) as a fluorescent OCT2 tracer substrate. All OCT2 substrates *cis*-inhibited DiASP uptake in OCT2-overexpressing HEK293 cells, with IC_50_ values ranging from 0.24 µM (for ipratropium) to 2.39 mM (for dopamine). By contrast, only 4/15 substrates, i.e., acetylcholine, agmatine, choline and metformin, *trans*-stimulated DiASP uptake, with a full suppression of the *trans*-stimulating effect of metformin by the reference OCT2 inhibitor amitriptyline. An analysis of molecular descriptors next indicated that *trans*-stimulating OCT2 substrates exhibit lower molecular weight, volume, polarizability and lipophilicity than non-*trans*-stimulating counterparts. Overall, these data indicated a rather low sensitivity (26.7%) of the *trans*-stimulation assay for identifying OCT2 substrates, and caution with respect to the use of such assay may therefore be considered.

## 1. Introduction

Solute carriers (SLCs) constitute a superfamily of more than 450 membrane proteins, acting as transporters of a large spectrum of molecules, including nutrients, metabolites, xenobiotics (such as phytochemicals), small molecule drugs and metal ions [1]. SLCs handling drugs are notably expressed by detoxifying organs, such as the liver and the kidney, and have been shown to play a major role in pharmacokinetics, drug–drug interactions and drug toxicity [2,3,4]. Some of them have consequently to be regulatorily studied during the pharmaceutical development of new molecular entities [5]. This is notably the case for organic cation transporter (OCT) 2 (*SLC22A2*), a uniporter expressed at the basolateral pole of proximal tubular cells [4]. The human OCT2 gene is located on chromosome 6 and contains 11 exons [6]. OCT2 has 12 predicted membrane-spanning domains and exhibits three glycosylated sites [7]. The driving force for this bi-directional facilitative diffusional transporter is believed to be the electrochemical gradient of the transported compounds [8]. Consequently, in cells with normal inside-negative membrane potential, cation uptake is energetically preferred, whereas cation efflux can only occur in depolarized cells or in the presence of a large outwardly directed cation gradient that overcomes the membrane potential [9]. In the kidney, OCT2 mediates uptake of cationic drugs such as metformin and cimetidine from the blood into renal cells [10]; it functions in conjunction with multidrug and toxin extrusion protein (MATE) 1 (*SLC47A1*) and MATE2-K (*SLC47A2*), which expel OCT2 substrates into the urine at the apical pole of proximal cells [11]. In this way, OCT2 plays a major role in the renal secretion of cationic drugs [12]; it is also involved in the nephrotoxicity of cisplatin [13]. For anionic drugs, organic anion transporter (OAT) 1 (*SLC22A6*) and OAT3 (*SLC22A8*), also expressed at the basolateral pole of renal proximal cells, such as OCT2, are implicated in their renal secretion [14].

Identification of substrates for SLCs constitutes an important issue. This is commonly addressed through comparing uptake of putative substrates in cellular models overexpressing or not the transporter of interest, in the absence or presence of specific transporter inhibitors [15]. Such studies require precisely quantifying the cellular accumulation of investigated compounds, through scintillation counting (if the compound is radiolabelled) or through sensitive liquid chromatography-tandem mass spectrometry (LC-MS/MS). Such approaches can however be extremely consuming either in time, money, or both, and may be difficult to integrate into high throughput processes. The use of *trans*-stimulation assays may represent an interesting alternative for the identification of SLC substrates, without the need of measuring intracellular concentrations of the candidate substrates [16]. In these assays, substrates are commonly identified by their ability to (1) *cis*-inhibit accumulation of a reference tracer substrate of the SLC and (2) *trans*-stimulate uptake or efflux of the reference tracer substrate [17,18,19]. The *trans*-stimulation process is based on the hypothesis that the rate constant for reorientation of a loaded carrier is faster than that for the empty, in particular for uniporters such as OCT2 [7]. For uptake *trans*-stimulation assays, SLC-overexpressing cells are first loaded with the candidate substrate; after washing, they are secondly incubated with the reference tracer substrate, in a candidate substrate-free medium. If the loaded compound is a substrate, it is postulated to be reverse-transported outside the cells by the SLC during the second phase of the assay. This results in a conformational switch of the transporter toward a state receptive to the handling of substrates at the extracellular face of the membrane, which finally causes an increased uptake of the tracer substrate [20]. For efflux *trans*-stimulation assays, SLC-overexpressing cells are first incubated with the reference tracer substrate, and, after washing, they are next incubated with the candidate substrate in reference tracer-free medium; the SLC-mediated transport of the candidate compound inside the cells is finally associated with an increased SLC-mediated efflux of the tracer substrate [21,22]. A variant of the efflux *trans*-stimulation assay corresponds to competitive counterflow, in which the reference tracer substrate remains present at the same concentration in the extracellular medium during both the loading and the efflux phases [23,24]. Finally, it is noteworthy that *trans*-stimulation assays can be performed with SLC-expressing membrane vesicles instead of cells [25]. Thus, rat renal membrane vesicles have been used for investigating *trans*-stimulation of peptide transporter (PEPT) activity by β-lactam antibiotics [26] and for studying basolateral OCT activity [27]. *trans*-stimulation assays have also been performed in situ at the mouse blood–brain barrier [28] and blood–retinal barrier [29].

*Trans*-stimulation assays have permitted to characterize substrates of various SLCs handling drugs, including OCT1 (*SLC22A1*) [30], OCT2 [16], MATE1 [22], OAT1 [18,31], organic anion transporting polypeptide (OATP) 2B1 (*SLCO2B1*) [24], sodium-taurocholate cotransporting polypeptide (NTCP/*SLC10A1*) [32] and an influx drug/proton antiporter expressed at the blood-brain barrier [33]. The sensitivity of *trans*-stimulation assays, i.e., their ability to correctly identify substrates, remains however yet rather elusive, although likely important to consider for the use of *trans*-stimulations assays. To get insights about this point in the present study, we have analysed the *trans*-stimulating properties of 15 structurally-diverse substrates of OCT2 towards transport of the OCT2 tracer substrate 4-(4-(dimethylamino)styryl)-N-methylpyridinium iodide (DiASP) in OCT2-overexpressing HEK293 cells. Our data demonstrated that, if all OCT2 substrates (*n* = 15) displayed *cis*-inhibitory effects towards OCT2 activity, only a few of them (*n* = 4) *trans*-stimulated OCT2-mediated uptake of DiASP, suggesting, therefore, a rather low sensitivity of this *trans*-stimulation assay for the identification of OCT2 substrates. 

## 2. Results

### 2.1. Cis-Inhibition of OCT2 Activity by OCT2 Substrates

**Figure 1:**
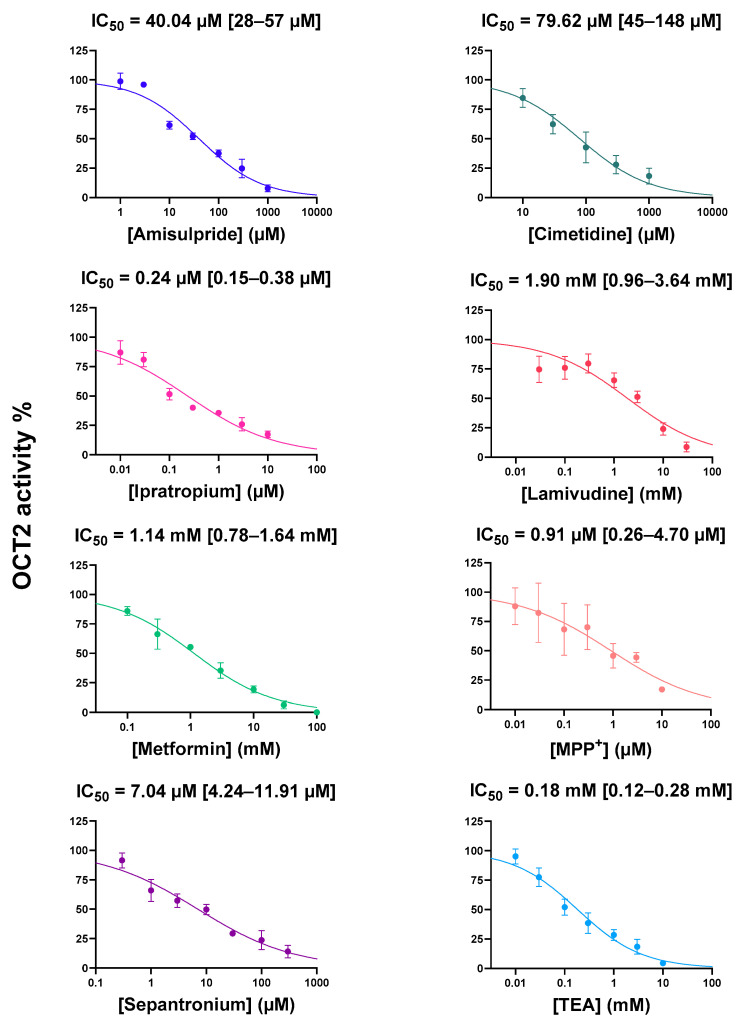
*Cis*-inhibition of OCT2 activity by exogenous OCT2 substrates. HEK-OCT2 cells were incubated with 10 µM DiASP, used here as a tracer substrate for OCT2, for 5 min at 37 °C, in the absence or presence of various concentrations of exogenous OCT2 substrates. Intracellular accumulation of DiASP was next determined by spectrofluorimetry and normalized to protein content. Data are expressed as % of OCT2 activity found in untreated control cells, arbitrarily set at 100%; they are the means ± SEM of at least three independent experiments performed in quadruplicate. IC_50_ values and 95% confidence intervals are indicated on the top of graphs.

**Figure 2:**
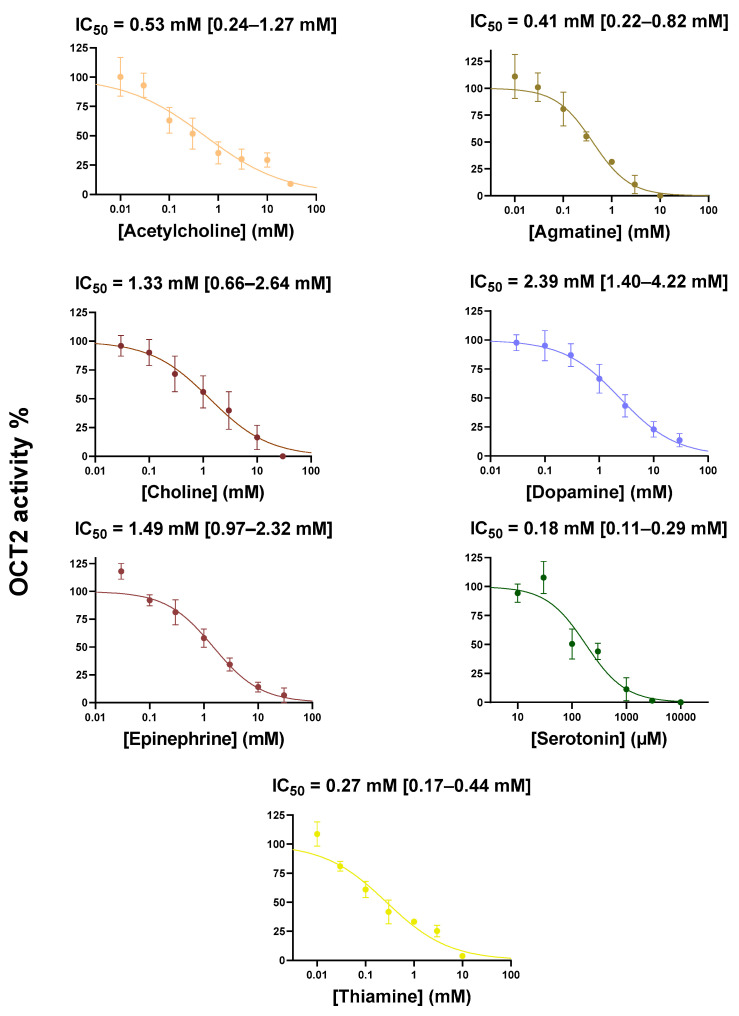
*Cis*-inhibition of OCT2 activity by endogenous OCT2 substrates. HEK-OCT2 cells were incubated with 10 µM DiASP, used here as a tracer substrate for OCT2, for 5 min at 37 °C, in the absence or presence of various concentrations of endogenous OCT2 substrates. Intracellular accumulation of DiASP was next determined by spectrofluorimetry and normalized to protein content. Data are expressed as % of OCT2 activity found in untreated control cells, arbitrarily set at 100%; they are the means ± SEM of at least three independent experiments performed in quadruplicate. IC_50_ values and 95% confidence intervals are indicated on the top of graphs.

**Figure 3:**
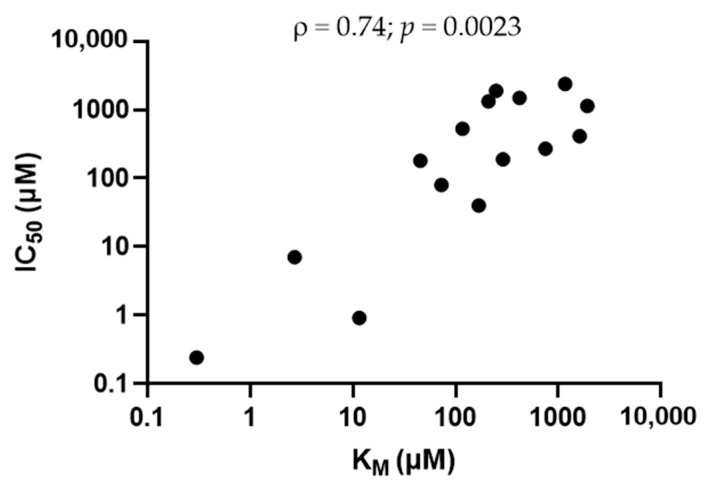
Spearman’s rank correlation between Michaelis constant (K_M_) and IC_50_ values for OCT2 substrates (*n* = 15). Spearman ρ coefficient and *p* value are indicated on the top of graph. K_M_ were from Table 1; K_M_ means were used for compounds for which various K_M_ have been reported.

Fifteen known OCT2 substrates, corresponding to laboratory/chemical reagents (*n* = 2), drugs (*n* = 6) and endogenous compounds/metabolites (*n* = 7) and whose affinities/Michaelis constant (K_M_) values for OCT2 are listed in Table 1, were investigated for their potential *cis*-inhibitory effects towards OCT2-mediated transport of DiASP. Beforehand, these OCT2 substrates have been demonstrated to not significantly impair DiASP-related fluorescence (Figure S1). As indicated in Figure 1, all exogenous substrates for OCT2, i.e., tetra-ethylammonium (TEA), 1-methyl-4-phenyl pyridinium (MPP^+^) and drugs, were found to *cis*-inhibit OCT2 activity, with half-maximal inhibitory concentrations (IC_50_) widely ranging from 0.24 µM (for ipratropium) to 1.90 mM (for lamivudine). Similarly, endogenous OCT2 substrates *cis*-inhibited OCT2 activity, with IC_50_ ranging from 0.18 mM (for serotonin) to 2.39 mM (for dopamine) (Figure 2). These IC_50_ values did not statistically differ between endogenous and exogenous substrates (*p* = 0.21). They were significantly positively correlated with K_M_ values for OCT2 (Figure 3), indicating that IC_50_ values were related to affinities of substrates for OCT2.

### 2.2. Trans-Stimulation of OCT2 Activity by OCT2 Substrates

**Figure 4:**
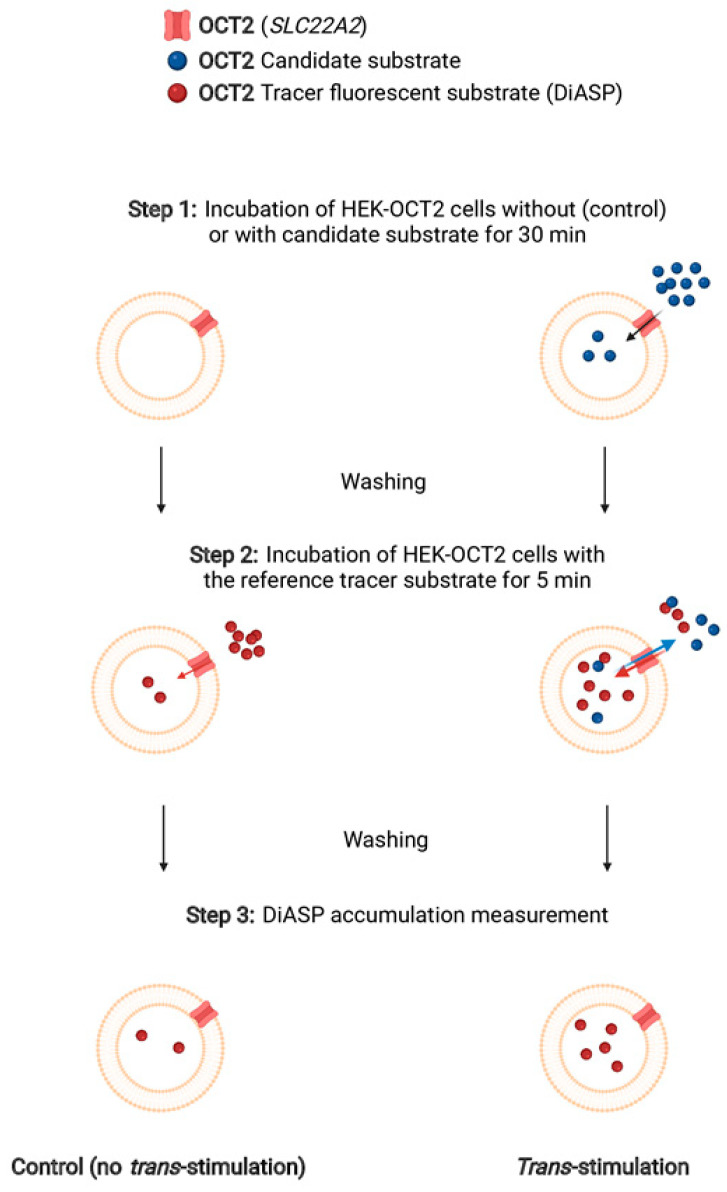
Schematic representation of the various steps of the *trans*-stimulation assay performed for investigating OCT2 substrates (created with biorender.com). Step 1 corresponds to the initial incubation of cells without (Control) or with candidate substrate for 30 min at 37 °C. Step 2 consists of the incubation of cells with the reference tracer substrate (DiASP) for 5 min at 37 °C. Step 3 is the measurement of cellular accumulation of DiASP through spectrofluorimetry.

**Figure 5:**
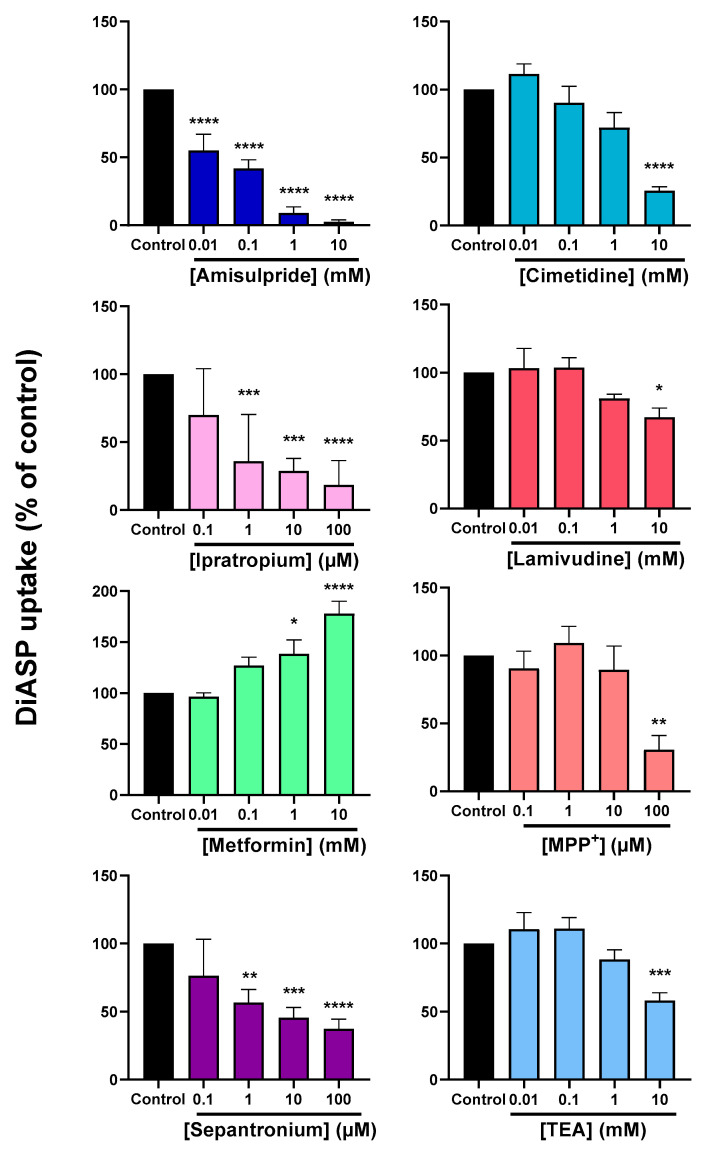
*Trans*-modulation of OCT2 activity by exogenous OCT2 substrates. HEK-OCT2 cells were first incubated in the absence (control) or presence of various concentrations of exogenous OCT2 substrates for 30 min at 37 °C. After washing, cells were next incubated with 10 µM DiASP, used here as a tracer substrate for OCT2, for 5 min at 37 °C. Accumulation of DiASP was finally determined by spectrofluorimetry and normalised to protein content. Data are expressed as % of DiASP accumulation found in untreated control cells, arbitrarily set at 100%; they are the means ± SEM of at least three independent experiments performed in quadruplicate. *, *p* < 0.05, **, *p* < 0.01, ***, *p* < 0.001 and ****, *p* < 0.0001, when compared to control.

**Figure 6:**
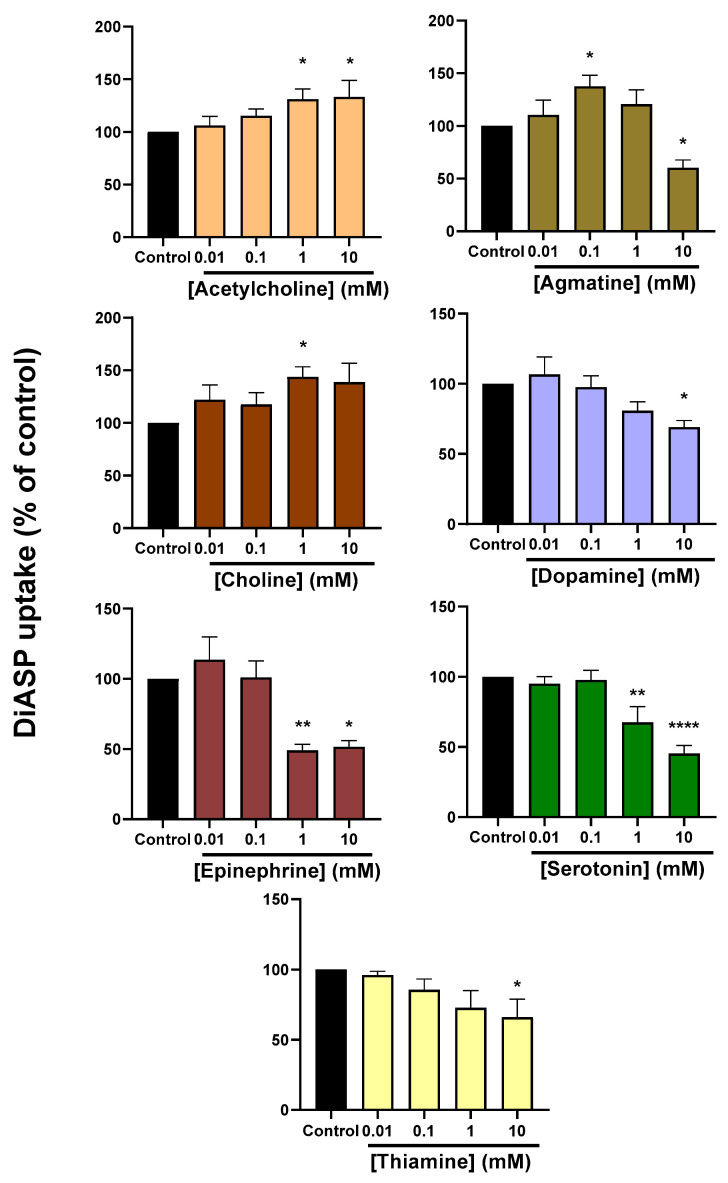
*trans*-modulation of OCT2 activity by endogenous OCT2 substrates. HEK-OCT2 cells were first incubated in the absence (control) or presence of various concentrations of endogenous OCT2 substrates for 30 min at 37 °C. After washing, cells were next incubated with 10 µM DiASP, used here as a tracer substrate for OCT2, for 5 min at 37 °C. Accumulation of DiASP was finally determined by spectrofluorimetry and normalised to protein content. Data are expressed as % of DiASP accumulation found in untreated control cells, arbitrarily set at 100%; they are the means ± SEM of at least three independent experiments performed in quadruplicate. *, *p* < 0.05, **, *p* < 0.01 and ****, *p* < 0.0001, when compared to control.

Uptake *trans*-stimulation assays were performed as schematically depicted in Figure 4. HEK-OCT2 cells were first exposed to different increasing concentrations of OCT2 substrates, usually in the 0.01–10 mM range, except for ipratropium, MPP^+^ and sepantronium, for which lower concentrations were used (0.1–100 µM), owing to their high affinities (low K_M_ and IC_50_ values) for OCT2 (Table 1 and Figure 1); after washing, cells were secondly exposed to the tracer substrate DiASP. Among exogenous substrates of OCT2, only metformin was found to *trans*-stimulate uptake of DiASP, when used at 1 or 10 mM (Figure 5). By contrast, the other drugs *trans*-inhibited DiASP uptake; however, the lowest *trans*-inhibiting concentrations differed according to OCT2 substrates, ranging from 1 µM (for ipratopium and sepantronium) to 10 mM (for TEA, cimetidine and lamivudine) (Figure 5). For endogenous substrates of OCT2, acetylcholine (1 and 10 µM), agmatine (0.1 mM) and choline (1 mM) were found to significantly *trans*-stimulate OCT2 activity (Figure 6). Agmatine, however, also *trans*-inhibited OCT2 activity when used at 10 mM (Figure 6), indicating that this compound may act as a bifunctional modulator of OCT2 activity, i.e., it may act as a *trans*-stimulator or a *trans*-inhibitor according to the used concentration. Other endogenous compounds were demonstrated to *trans*-inhibit OCT2-mediated uptake of DiASP, with the lowest *trans*-inhibitory concentrations ranging from 1 mM (for epinephrine and serotonin) to 10 mM (for dopamine and thiamine) (Figure 6).

**Figure 7:**
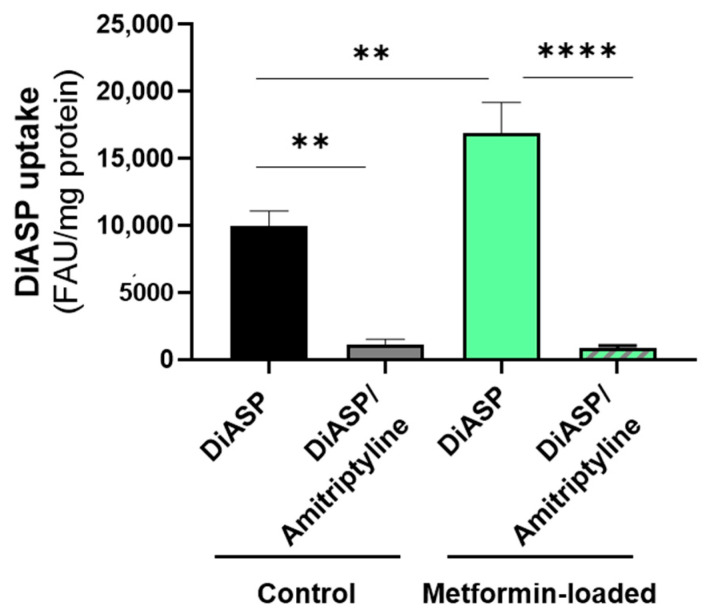
Effects of the reference OCT2 inhibitor amitriptyline on metformin-mediated *trans*-stimulation of DiASP uptake in HEK-OCT2 cells. HEK-OCT2 cells were either untreated (control) or treated by 10 mM metformin for 30 min at 37 °C. After washing, cells were incubated with 10 µM DiASP for 5 min at 37 °C, in the absence or presence of 100 µM amitriptyline. Accumulation of DiASP was finally determined by spectrofluorimetry and normalised to protein content. Data are expressed as DiASP-related fluorescence arbitrary unit (FAU)/mg protein and are the means ± SEM of at least three independent experiments performed in quadruplicate. **, *p* < 0.01 and ****, *p* < 0.0001.

For further investigating whether the *trans*-stimulating effects of OCT2 substrates towards OCT2 activity were formally linked to OCT2, we next analysed the effects of the reference OCT2 inhibitor amitriptyline [50] towards metformin-mediated *trans*-stimulation of DiASP uptake in HEK-OCT2 cells. As indicated in Figure 7, amitriptyline fully suppressed metformin-stimulated uptake of DiASP; uptake of DiASP in HEK-OCT2 cells not preloaded with metformin was also abolished. Moreover, metformin failed to significantly *trans*-stimulate DiASP uptake in control HEK-MOCK cells (data not shown). 

### 2.3. Physico-Chemical Parameters Associated with Trans-Stimulation of OCT2 Activity by OCT2 Substrates

Among fifteen known OCT2 substrates, only four, i.e., acetylcholine, agmatine, choline and metformin, were found to significantly *trans*-stimulate OCT2-mediated DiASP uptake in HEK-OCT2 cells, which indicates a rather low sensitivity (26.7%) of the *trans*-stimulation assay for correctly identifying OCT2 substrates. To determine whether *trans*-stimulating OCT2 substrates may exhibit specific physico-chemical features comparatively to non-*trans*-stimulating counterparts, we compared molecular descriptor values as well as those of K_M_ and IC_50_ (affinity parameters for OCT2), between *trans*-stimulating OCT2 substrates (*n* = 4) and non-*trans*-stimulating ones (*n* = 11). As shown in Table 2, 30/82 molecular descriptors exhibit significant differences between *trans*-stimulating and non-*trans*-stimulating OCT2 substrates. Analysis of these descriptors/parameters indicated that *trans*-stimulating compounds exhibit notably lower lipophilicity (ALOGP and MLOGP), volume (Mv, Sv, Vx and VvdwMG), molecular weight (MW), aromatic bond number (nAB) and polarizability (Mp and Sp) when compared to non-*trans*-stimulating ones; they additionally show increased first ionisation potential (Mi) (Table 2). With respect to affinity parameters (K_M_ and IC_50_), they do not statistically discriminate between *trans*-stimulating and non-*trans*-stimulating OCT2 substrates (data not shown), even if a trend to higher K_M_ values and thus lower affinities is observed for *trans*-stimulating compounds versus non-*trans*-stimulating ones (*p* = 0.056).

## 3. Discussion

*Trans*-stimulation assays have been presumed to represent an easy and rapid way for the identification of drugs substrates for SLCs, notably allowing to discard the use of radiolabelled molecules or the fastidious development of LC-MS/MS methods for molecule dosages. We however reported a rather low sensitivity (26.7%) of *trans*-stimulation assays for the identification of OCT2 substrates in the present study, using a set of OCT2 substrates (*n* = 15) displaying various chemical structures and affinities for OCT2. Indeed, only 4/15 OCT2 substrates were found to both *cis*-inhibit and *trans*-stimulate OCT2 activity, which is usually required for considering them as substrates. The other 11/15 OCT2 substrates both *cis*- and *trans*-inhibited OCT2 activity, confirming thus that they also interacted with OCT2, but without evidence for being transported. Indeed, even if the fact that these non-*trans*-stimulating compounds exert *trans*-inhibitory effects may be considered as indirect proof of their putative transport into cells, this cellular uptake does not formally necessarily be linked to OCT2 and may notably reflect passive transport across the plasma membrane [51]. The fact that these non-*trans*-stimulating substrates display notable lipophilicity (ALOGP and MLOGP) may agree with this hypothesis. 

Even if restricted to only 4/15 compounds, *trans*-stimulatory effects are most likely related to OCT2 activity, notably for metformin, because (i) the reference OCT2 inhibitor amitriptyline fully abrogated metformin-mediated *trans*-stimulation of DiASP in HEK-OCT2 cells and (ii) the antidiabetic drug *trans*-stimulated DiASP uptake in HEK-OCT2 cells, but not in control HEK-MOCK cells. Therefore, these data clearly support the concept of *trans*-stimulation for OCT2, even if it can be not extended to all substrates. In this context, choline and metformin have already been shown to *trans*-stimulate OCT2 activity using radiolabelled MPP^+^ as a tracer substrate [23], thus confirming that these compounds are likely robust *trans*-stimulating agents of OCT2 activity. In the same way, agmatine has been demonstrated to *trans*-stimulate the activity of OCT3 (SLC22A3) [16], thus suggesting that this compound is a global *trans*-stimulator of OCTs. Furthermore, OCT2 can mediate cellular efflux of acetylcholine [34], which is fully consistent with the OCT2 *trans*-stimulatory effects of this neurotransmitter; indeed, enhanced uptake of the tracer substrate in *trans*-stimulation assays is presumed to be triggered by efflux of the *trans*-stimulating agent and the resulting conformational switch of the loaded transporter, resulting in an extracellular-facing binding site of the transporter [7].

The reason for which a majority of OCT2 substrates (11/15) failed to *trans*-stimulate OCT2-mediated uptake of DiASP activity remains to be determined. This is particularly intriguing for TEA, which has been previously found to *trans*-stimulate OCT2 activity, with however the use of radiolabelled MPP^+^ or TEA as tracer reference substrates, indeed of that of DiASP [20,23,52]. The nature of the reference tracer substrate used in *trans*-stimulation assays may therefore be hypothesized to contribute to the discrepancy with respect to TEA *trans*-stimulating effects towards OCT2 activity, as already been demonstrated for OCT2 *cis*-inhibition studies, in which the inhibitory potential of various drugs depends on the nature of the probe substrate [53]. Moreover, MPP^+^ and cimetidine failed to *trans*-stimulate, not only OCT2-mediated DiASP uptake but also that of MPP^+^ [20], confirming therefore that these OCT2 substrates exhibit rather poor *trans*-stimulation potential in uptake studies. This lack of *trans*-stimulation caused by these compounds and the other non-*trans*-stimulating compounds is likely not due to the use of inappropriate concentrations of them in *trans*-stimulation assays. Indeed, a large range of concentrations has been tested for each compound, including high concentrations being at least fourfold, and often more than tenfold greater than IC_50_ values, and thus in the range of minimal concentrations usually recommended in *trans*-stimulation assays [22,54].

Low-affinity substrates of OCT2 have been hypothesized to have greater *trans*-stimulating potential when compared to high-affinity substrates, due to their higher rates of dissociation from OCT2 [20]. Indeed, the rate-limiting step in OCT2-mediated transport may be the dissociation of the substrate from the transporter, rather than differences in conformational change of the transport protein associated with the translocation of structurally distinct substrates [7]. However, neither K_M_ values nor IC_50_ were found to statistically discriminate between *trans*-stimulating and non-*trans*-stimulating OCT2 substrates in the present study, even if a trend exists for K_M_ values. In particular, if the 4 *trans*-stimulating compounds display rather high K_M_ values (in the 117–3171 µM range), non-*trans*-stimulating substrates such as dopamine or thiamine also exhibit similar high K_M_ values, i.e., 932–1400 µM for dopamine and 750 µM for thiamine (Table 1). Taken together, these data do not highlight affinity as a major discriminating factor between *trans*-stimulating or non-*trans*-stimulating OCT2 substrates. By contrast, various molecular descriptors were found to be significantly associated with a *trans*-stimulating activity of OCT2 substrates. *Trans*-stimulating OCT2 substrates thus appear to exhibit lower molecular weight, volume, polarizability and lipophilicity than non-*trans*-stimulating counterparts. The parameter lipophilicity likely deserves special attention, because it can be hypothesized that non-*trans*-stimulating compounds, which are more lipophilic than *trans*-stimulating counterparts, may at least partly cross back the plasma membrane by passive diffusion after their loading, without mobilizing OCT2 in a major way and therefore without triggering the conformational switch of the transporter required for the *trans*-stimulation process. Moreover, the resulting extracellular concentrations of passive diffusion-based transport for these non-*trans*-stimulating substrates may rapidly rise to levels sufficient for inhibiting OCT2 activity; this may explain the observed *trans*-inhibitory effects of these compounds when they are loaded at high concentrations. This hypothesis is likely supported by the fact that the OAT3 substrate estrone-sulfate has been found to *trans*-inhibit OAT3 activity, likely due to competition with the tracer substrate for re-entry at the *ci*s-side [55]. Such a re-entry process may also contribute to the biphasic effect of agmatine, with *trans*-stimulation of OCT2 activity for a low loading concentration and *trans*-inhibition at higher loading concentration, possibly due to the fact that effluxed agmatine reached extracellular levels allowing competition with DiASP for OCT2-mediated entry into cells. As an alternative to competition for re-entry, *trans*-inhibition may be due to binding to intracellular regulatory sites of OCT2 by *trans*-inhibiting agents, knowing that OCT2 substrates are thought to bind to multiple non-overlapping sites on OCT2 [7]. Finally, side-specificity of the transport as well different affinities of extracellular and intracellular binding sites of OCT2 for drugs may also be considered for putatively explaining the lack of trans-stimulation effect of 11/15 substrates. The possible existence of multiple drug binding sites on OCT2 with variable affinities may argue in favour of this hypothesis [9]. Additional studies, notably molecular docking analyses, may help to better understand why some OCT2 substrates fail to *trans*-stimulate OCT2-mediated DiASP transport.

The low level of sensitivity of OCT2 activity *trans*-stimulation assays for the identification of substrates, i.e., the notable number of false negatives does not argue in favour of the general use of such assays performed in the experimental conditions described in the present study. Overall, this also highlights the rather complexity of the *trans*-stimulation process, however already applied to the study of SLCs handling glucose or amino acids [56,57]. However, it remains noteworthy that *trans*-stimulation was demonstrated to be well-operating for certain OCT2 substrates such as metformin, with a fluorescence dye as a tracer substrate. This last point has to be underlined, since the use of fluorochromes in *trans*-stimulation studies may be applicable to high throughput studies, as already described for *cis*-inhibition analyses of transporters [58]. Further studies are likely required to search improved experimental conditions for *trans*-stimulation assays, including the development of efflux *trans*-stimulation or competitive counterflow approaches, in order to enhance their sensitivity for identifying substrates, notably for organic cation transporters. The specificity of these *trans*-stimulation assays, i.e., the rate of false positives would be complementary important to investigate; comparison of the sensitivities of *trans*-stimulation assays based on fluorescent tracer substrates with those using radiolabelled tracer substrates or tracers investigated by LC-MS/MS would be also worthy of interest.

In summary, 15 various OCT2 substrates were found to all *cis*-inhibited OCT2-mediated uptake of the fluorescent dye DiASP, but only a few of them (4/15), including the reference substrate metformin, *trans*-stimulated it. Caution has therefore likely to be considered when using such *trans*-stimulation assays for the identification of drug substrates for SLCs such as OCT2.

## 4. Materials and Methods

### 4.1. Chemicals

The exogenous OCT2 substrates amisulpride, cimetidine, ipratropium, lamivudine, metformin, MPP^+^, TEA and sepantronium (also known as YM155), as well as the endogenous OCT2 substrates acetylcholine, agmatine, choline, dopamine, epinephrine, serotonin and thiamine and the OCT2 inhibitor amitriptyline were provided by Sigma-Aldrich (Saint Quentin Fallavier, France). The chemical structures of the OCT2 substrates are indicated in Figure S2. The fluorescent dye DiASP, used here as a reference tracer substrate for OCT2 [59,60], was purchased from Invitrogen (Carlsbad, CA, USA). 

### 4.2. Cell Culture

Human embryonic kidney HEK293 cells overexpressing human OCT2 (HEK-OCT2 cells) and corresponding control HEK-MOCK cells were provided by Vectalys (Labege, France) and have been previously functionally characterized [61]. They were cultured in Dulbecco’s modified Eagle medium (DMEM) (Life Technologies, Carlsbad, CA, USA), supplemented with 10% (vol/vol) fetal calf serum, 10 IU/mL penicillin, 10 μg/mL streptomycin, 1% non-essential amino acids, and 1 μg/mL insulin, as already reported [52].

### 4.3. OCT2 Activity Assays

#### 4.3.1. *Cis*-Inhibition Assays

*Cis*-inhibition of OCT2 activity was analysed as previously described [52]. Briefly, HEK-OCT2 cells were incubated with 10 µM DiASP, in the absence (control) of the presence of 100 µM amitriptyline (used here as a reference inhibitor of OCT2 activity) or of various concentrations of the OCT2 substrates, for 5 min. The transport assay medium consisted of 5.3 mM KCl, 1.1 mM KH_2_PO_4_, 0.8 mM MgSO_4_, 1.8 mM CaCl_2_, 11 mM dextrose, 10 mM HEPES, and 136 mM NaCl, adjusted to pH = 7.4. Next, cells were washed with phosphate-buffered saline (PBS) and lysed in distilled water. Intracellular accumulation of DiASP was then determined by spectrofluorimetry using a PerkinElmer Enspire spectrofluorometer (Waltham, MA, USA); excitation and emission wavelengths were 485 nm and 607 nm, respectively. DiASP accumulation values were thereafter normalized to total protein content, determined by the Bradford method [62]. Data were expressed as percentages of transporter activity found in control cells, arbitrarily set at 100%, according to the following Equation (1):

(1) $$\mathrm{OCT}2\;\mathrm{activity}\;\%\;=\;\frac{\left[{\mathrm{DiASP}}_{\mathrm{OCT}2\;\mathrm{substrate}}\right]\;-\;\left[{\mathrm{DiASP}}_{\mathrm{Amitriptyline}}\right]}{\left[{\mathrm{DiASP}}_{\mathrm{Control}}]-[{\mathrm{DiASP}}_{\mathrm{Amitriptyline}}\right]}\;\times \;100\;$$

where [DiASP_OCT2_ _substrate_] = DiASP accumulation in the presence of a defined concentration of OCT2 substrate, [DiASP_Amitriptyline_] = DiASP accumulation in the presence of amitriptyline and [DiASPControl] = DiASP accumulation in untreated control cells.

IC_50_ values of OCT2 substrates towards OCT2-mediated uptake of DiASP and the corresponding 95% confidence intervals were determined from nonlinear regression of concentration-response data, based on the four-parameter logistic function. They were calculated using Prism 9.3 software (GraphPad Software, San Diego, CA, USA), through the following Equation (2):

(2) $$A\;=\;\frac{100}{1\;+\;10^{\left(\left[\mathrm{OCT}2\;\mathrm{substrate}\right]\;-\;{\mathrm{LogIC}}_{50}\right)\;\times \;\mathrm{Hill}\;\mathrm{slope}}}\;$$

where A = the percentage of transporter activity for a given concentration of OCT2 substrate determined as described in Equation (1), [OCT2 substrate] = OCT2 substrate concentration in the transport assay medium, and Hill slope = a coefficient describing the steepness of the curve.

#### 4.3.2. *Trans*-Stimulation Assays

OCT2 *trans*-stimulation assays were performed through monitoring cellular uptake of the tracer substrate DiASP. As schematically depicted in Figure 4, HEK-OCT2 cells were first incubated in the absence (control) or presence of different concentrations of OCT2 substrates for 30 min at 37 °C in the transport assay medium described above. After washing with PBS, cells were re-incubated with 10 µM DiASP for 5 min. Cellular accumulation of the fluorescence dye was next determined by spectrofluorimetry, as reported above. Data were commonly expressed as % of DiASP uptake in control cells, set at 100%, or as fluorescence arbitrary units (FAU)/mg protein.

### 4.4. Molecular Descriptors Generation

Eighty-two molecular descriptors listed in Table S1 and belonging to the blocks “constitutional indices” (*n* = 47), “charge descriptors” (*n* = 15), and “molecular properties” (*n* = 20), were determined for each of the 15 OCT2 substrates investigated in the study, using the Dragon 7.0 software (Talete, Milano, Italy). OCT2 substrates, initially expressed in SMILES format, were converted to 3D format using MarvinView 20.20.0 software (ChemAxon, Budapest, Hungary) before processing by Dragon 7.0 software to obtain molecular descriptors, as previously described [52,63]. 

### 4.5. Data Analysis

Experimental data are usually expressed as means ± SEM from at least three independent experiments, each being performed in quadruplicate. They were statistically analyzed using Prism 9.3 software through analysis of variance (ANOVA) followed by Dunnett’s or Bonferroni’s post hoc test. The relationship between measured IC_50_ and K_M_ values reported in the literature for each OCT2 substrate was analysed through the nonparametric Spearman’s rank correlation method. Comparison of molecular descriptor values between *trans*-stimulating and non-*trans*-stimulating OCT2 substrates was performed using the *t*-test, which is applicable to small sample sizes [64]. The criterion of significance for statistical tests was *p* < 0.05. The sensitivity of the *trans*-stimulation assay for identifying OCT2 substrates was calculated according to the following equation:

(3) $$\mathrm{Sensitivity}\;=\;\frac{\mathrm{Number}\;\mathrm{of}\;\mathrm{OCT}2\;\mathrm{substrates}\;cis\;-\;\mathrm{inhibiting}\;\mathrm{and}\;trans\;-\;\mathrm{stimulating}\;\mathrm{OCT}2\;\mathrm{activity}}{\mathrm{Number}\;\mathrm{of}\;\mathrm{OCT}2\;\mathrm{substrates}}\;\times \;100$$

## Figures and Tables

**Table 1:** Affinity for OCT2 (K_M_ values) of substrates used in the study.

OCT2 Substrate	Nature	K_M_ (µM)	Reference
Acetylcholine	Endogenous substance (neurotransmitter)	117	[34]
Agmatine	Endogenous substance (metabolite)	1400–1840	[16,35]
Amisulpride	Exogenous substance (antiemetic drug)	168	[36]
Choline	Endogenous substance (neurotransmitter)	210	[37]
Cimetidine	Exogenous substance (antihistamine drug)	73	[38]
Dopamine	Endogenous substance (neurotransmitter)	932–1400	[39,40]
Epinephrine	Endogenous substance (biogenic amine)	420	[40]
Ipratropium	Exogenous substance (anticholinergic drug)	0.3	[41]
Lamivudine	Exogenous substance (antiretroviral drug)	248	[42]
Metformin	Exogenous substance (antidiabetic drug)	990–3171	[39,43,44]
MPP^+^	Exogenous substance (laboratory reagent)	3.1–20	[39,45,46]
Sepantronium	Exogenous substance (anticancer drug)	2.7	[47]
Serotonin	Endogenous substance (neurotransmitter)	290	[40]
TEA	Exogenous substance (laboratory reagent)	28.5–76	[37,48]
Thiamine	Endogenous substance (vitamin precursor)	750	[49]

**Table 2:** Molecular descriptors significantly discriminating OCT2 substrates according to their *trans*-stimulation properties towards OCT2-mediated DiASP uptake. CI, constitutional indices; MP, molecular properties; CD, charge descriptors.

Molecular Descriptor			Value Mean		*p*-Value (*t*-Test)
			OCT2 Substrates		
Block	Name	Description	*Trans*-Stimulating	Non-*Trans*-Stimulating	
CI	Mi	Mean first ionization potential (scaled on Carbon atom)	1.17	1.14	0.0004
CI	Mp	Mean atomic polarizability (scaled on Carbon atom)	0.56	0.64	0.0007
MP	ALOGP	Ghose-Crippen octanol-water partition coeff. (logP)	−1.02	0.61	0.0012
CI	C%	Percentage of C atoms	23.10	35.93	0.0015
CI	Mv	Mean atomic van der Waals volume (scaled on Carbon atom)	0.51	0.60	0.0030
MP	Uc	Unsaturation count	0.90	2.82	0.0054
CI	GD	Graph density	0.23	0.15	0.0061
CI	nAB	Number of aromatic bonds	0	5.73	0.0073
MP	BLTF96	Verhaar Fish base-line toxicity from MLOGP (mmol/L)	0.04	−1.83	0.0079
MP	MLOGP	Moriguchi octanol-water partition coeff. (logP)	−1.68	0.52	0.0079
MP	BLTD48	Verhaar Daphnia base-line toxicity from MLOGP (mmol/L)	0.28	−1.81	0.0080
MP	BLTA96	Verhaar Algae base-line toxicity from MLOGP (mmol/L)	0.45	−1.74	0.0080
CI	nBM	Number of multiple bonds	1	7.36	0.0094
CI	nCsp2	Number of sp2 hybridized Carbon atoms	1	6.82	0.0120
CI	SCBO	Sum of conventional bond orders (H-depleted)	8.75	22.23	0.0123
MP	Ui	Unsaturation index	0.90	2.30	0.0130
CI	nC	Number of Carbon atoms	5.25	12.18	0.0131
CI	nBO	Number of non-H bonds	7.75	17.64	0.0161
CI	H%	Percentage of H atoms	61.03	50.29	0.0188
CD	RNCG	Relative negative charge	0.48	0.25	0.0195
CI	nSK	Number of non-H atoms	8.75	16.82	0.0225
CI	MW	Molecular weight	127.5	238.7	0.0254
CI	Sv	Sum of atomic van der Waals volumes (scaled on carbon atom)	11.49	20.28	0.0295
CI	Sp	Sum of atomic polarizabilities (scaled on carbon atom)	12.54	21.65	0.0325
CI	AMW	Average molecular weight	5.68	7.05	0.0350
MP	Vx	McGowan volume	188.5	309.8	0.0366
MP	VvdwMG	van der Waals volume from McGowan volume	80.62	130.3	0.0366
MP	AMR	Ghose–Crippen molar refractivity	35.26	62.75	0.0383
MP	VvdwZAZ	van der Waals volume from Zhao–Abraham–Zissimos equation	134.2	225	0.0395
MP	PDI	Packing density index	0.82	0.91	0.0418

## Data Availability

Data of the study are available from the corresponding author upon reasonable request.

## Supplementary Materials

The following are available online at www.mdpi.com/ccc/s1.

## References

[1] Pizzagalli M.D., Bensimon A., Superti-Furga G. (2021). A guide to plasma membrane solute carrier proteins. FEBS J..

[2] Giacomini K.M., Huang S.M., Tweedie D.J., Benet L.Z., Brouwer K.L., Chu X., Dahlin A., Evers R., Fischer V., Hillgren K.M. (2010). Membrane transporters in drug development. Nat. Rev. Drug Discov..

[3] Brecht K., Schäfer A.M., Meyer Zu Schwabedissen H.E. (2020). Uptake Transporters of the SLC21, SLC22A, and SLC15A Families in Anticancer Therapy-Modulators of Cellular Entry or Pharmacokinetics?. Cancers.

[4] Nies A.T., Koepsell H., Damme K., Schwab M. (2011). Organic cation transporters (OCTs, MATEs), in vitro and in vivo evidence for the importance in drug therapy. Handb. Exp. Pharmacol..

[5] Lee S.C., Arya V., Yang X., Volpe D.A., Zhang L. (2017). Evaluation of transporters in drug development: Current status and contemporary issues. Adv. Drug Deliv. Rev..

[6] Gründemann D., Schömig E. (2000). Gene structures of the human non-neuronal monoamine transporters EMT and OCT2. Hum. Genet..

[7] Wright S.H. (2019). Molecular and cellular physiology of organic cation transporter 2. Am. J. Physiol. Ren. Physiol..

[8] Budiman T., Bamberg E., Koepsell H., Nagel G. (2000). Mechanism of electrogenic cation transport by the cloned organic cation transporter 2 from rat. J. Biol. Chem..

[9] Koepsell H. (2019). Multiple binding sites in organic cation transporters require sophisticated procedures to identify interactions of novel drugs. Biol. Chem..

[10] Samodelov S.L., Kullak-Ublick G.A., Gai Z., Visentin M. (2020). Organic Cation Transporters in Human Physiology, Pharmacology, and Toxicology. Int. J. Mol. Sci..

[11] Motohashi H., Inui K. (2013). Multidrug and toxin extrusion family SLC47: Physiological, pharmacokinetic and toxicokinetic importance of MATE1 and MATE2-K. Mol. Asp. Med..

[12] Koepsell H. (2020). Organic Cation Transporters in Health and Disease. Pharmacol. Rev..

[13] Hucke A., Rinschen M.M., Bauer O.B., Sperling M., Karst U., Köppen C., Sommer K., Schröter R., Ceresa C., Chiorazzi A. (2019). An integrative approach to cisplatin chronic toxicities in mice reveals importance of organic cation-transporter-dependent protein networks for renoprotection. Arch. Toxicol..

[14] Burckhardt G. (2012). Drug transport by Organic Anion Transporters (OATs). Pharmacol. Ther..

[15] Dvorak V., Wiedmer T., Ingles-Prieto A., Altermatt P., Batoulis H., Bärenz F., Bender E., Digles D., Dürrenberger F., Heitman L.H. (2021). An Overview of Cell-Based Assay Platforms for the Solute Carrier Family of Transporters. Front. Pharmacol..

[16] Gründemann D., Hahne C., Berkels R., Schömig E. (2003). Agmatine is efficiently transported by non-neuronal monoamine transporters extraneuronal monoamine transporter (EMT) and organic cation transporter 2 (OCT2). J. Pharmacol. Exp. Ther..

[17] Dresser M.J., Xiao G., Leabman M.K., Gray A.T., Giacomini K.M. (2002). Interactions of n-tetraalkylammonium compounds and biguanides with a human renal organic cation transporter (hOCT2). Pharm. Res..

[18] Hagos Y., Schley G., Schödel J., Krick W., Burckhardt G., Willam C., Burckhardt B.C. (2012). α-Ketoglutarate-related inhibitors of HIF prolyl hydroxylases are substrates of renal organic anion transporters 1 (OAT1) and 4 (OAT4). Pflugers Arch..

[19] Blazquez A.G., Briz O., Romero M.R., Rosales R., Monte M.J., Vaquero J., Macias R.I., Cassio D., Marin J.J. (2012). Characterization of the role of ABCG2 as a bile acid transporter in liver and placenta. Mol. Pharmacol..

[20] Severance A.C., Sandoval P.J., Wright S.H. (2017). Correlation between Apparent Substrate Affinity and OCT2 Transport Turnover. J. Pharmacol. Exp. Ther..

[21] Bourdet D.L., Pritchard J.B., Thakker D.R. (2005). Differential substrate and inhibitory activities of ranitidine and famotidine toward human organic cation transporter 1 (hOCT1; SLC22A1), hOCT2 (SLC22A2), and hOCT3 (SLC22A3). J. Pharmacol. Exp. Ther..

[22] Martínez-Guerrero L.J., Wright S.H. (2013). Substrate-dependent inhibition of human MATE1 by cationic ionic liquids. J. Pharmacol. Exp. Ther..

[23] Harper J.N., Wright S.H. (2013). Multiple mechanisms of ligand interaction with the human organic cation transporter, OCT2. Am. J. Physiol. Ren. Physiol..

[24] Schäfer A.M., Bock T., Meyer Zu Schwabedissen H.E. (2018). Establishment and Validation of Competitive Counterflow as a Method To Detect Substrates of the Organic Anion Transporting Polypeptide 2B1. Mol. Pharm..

[25] McKinney T.D., Hosford M.A. (1992). Organic cation transport by rat hepatocyte basolateral membrane vesicles. Am. J. Physiol..

[26] Takahashi K., Nakamura N., Terada T., Okano T., Futami T., Saito H., Inui K.I. (1998). Interaction of beta-lactam antibiotics with H+/peptide cotransporters in rat renal brush-border membranes. J. Pharmacol. Exp. Ther..

[27] Takano M., Inui K., Okano T., Saito H., Hori R. (1984). Carrier-mediated transport systems of tetraethylammonium in rat renal brush-border and basolateral membrane vesicles. Biochim. et Biophys. Acta.

[28] Cisternino S., Chapy H., André P., Smirnova M., Debray M., Scherrmann J.M. (2013). Coexistence of passive and proton antiporter-mediated processes in nicotine transport at the mouse blood-brain barrier. AAPS J..

[29] Chapy H., André P., Declèves X., Scherrmann J.M., Cisternino S. (2015). A polyspecific drug/proton antiporter mediates diphenhydramine and clonidine transport at the mouse blood-retinal barrier. Br. J. Pharmacol..

[30] Dresser M.J., Gray A.T., Giacomini K.M. (2000). Kinetic and selectivity differences between rodent, rabbit, and human organic cation transporters (OCT1). J. Pharmacol. Exp. Ther..

[31] Apiwattanakul N., Sekine T., Chairoungdua A., Kanai Y., Nakajima N., Sophasan S., Endou H. (1999). Transport properties of nonsteroidal anti-inflammatory drugs by organic anion transporter 1 expressed in Xenopus laevis oocytes. Mol. Pharmacol..

[32] Müller J.P., Keufgens L., Gründemann D. (2021). Hyperosmolarity stimulates transporter-mediated insertion of estrone sulfate into the plasma membrane, but inhibits the uptake by SLC10A1 (NTCP). Biochem. Pharmacol..

[33] Chapy H., Goracci L., Vayer P., Parmentier Y., Carrupt P.A., Declèves X., Scherrmann J.M., Cisternino S., Cruciani G. (2015). Pharmacophore-based discovery of inhibitors of a novel drug/proton antiporter in human brain endothelial hCMEC/D3 cell line. Br. J. Pharmacol..

[34] Lips K.S., Volk C., Schmitt B.M., Pfeil U., Arndt P., Miska D., Ermert L., Kummer W., Koepsell H. (2005). Polyspecific cation transporters mediate luminal release of acetylcholine from bronchial epithelium. Am. J. Respir. Cell Mol. Biol..

[35] Winter T.N., Elmquist W.F., Fairbanks C.A. (2011). OCT2 and MATE1 provide bidirectional agmatine transport. Mol. Pharm..

[36] Dos Santos Pereira J.N., Tadjerpisheh S., Abu Abed M., Saadatmand A.R., Weksler B., Romero I.A., Couraud P.O., Brockmöller J., Tzvetkov M.V. (2014). The poorly membrane permeable antipsychotic drugs amisulpride and sulpiride are substrates of the organic cation transporters from the SLC22 family. AAPS J..

[37] Gorboulev V., Ulzheimer J.C., Akhoundova A., Ulzheimer-Teuber I., Karbach U., Quester S., Baumann C., Lang F., Busch A.E., Koepsell H. (1997). Cloning and characterization of two human polyspecific organic cation transporters. DNA Cell Biol..

[38] Tahara H., Kusuhara H., Endou H., Koepsell H., Imaoka T., Fuse E., Sugiyama Y. (2005). A species difference in the transport activities of H2 receptor antagonists by rat and human renal organic anion and cation transporters. J. Pharmacol. Exp. Ther..

[39] Zolk O., Solbach T.F., König J., Fromm M.F. (2009). Functional characterization of the human organic cation transporter 2 variant p.270Ala>Ser. Drug Metab. Dispos..

[40] Amphoux A., Vialou V., Drescher E., Brüss M., Mannoury La Cour C., Rochat C., Millan M.J., Giros B., Bönisch H., Gautron S. (2006). Differential pharmacological in vitro properties of organic cation transporters and regional distribution in rat brain. Neuropharmacology.

[41] Chen J., Brockmöller J., Seitz T., König J., Chen X., Tzvetkov M.V. (2017). Tropane alkaloids as substrates and inhibitors of human organic cation transporters of the SLC22 (OCT) and the SLC47 (MATE) families. Biol. Chem..

[42] Jung N., Lehmann C., Rubbert A., Knispel M., Hartmann P., van Lunzen J., Stellbrink H.J., Faetkenheuer G., Taubert D. (2008). Relevance of the organic cation transporters 1 and 2 for antiretroviral drug therapy in human immunodeficiency virus infection. Drug Metab. Dispos..

[43] Kimura N., Masuda S., Tanihara Y., Ueo H., Okuda M., Katsura T., Inui K. (2005). Metformin is a superior substrate for renal organic cation transporter OCT2 rather than hepatic OCT1. Drug Metab. Pharmacokinet..

[44] Elsby R., Chidlaw S., Outteridge S., Pickering S., Radcliffe A., Sullivan R., Jones H., Butler P. (2017). Mechanistic in vitro studies confirm that inhibition of the renal apical efflux transporter multidrug and toxin extrusion (MATE) 1, and not altered absorption, underlies the increased metformin exposure observed in clinical interactions with cimetidine, trimethoprim or pyrimethamine. Pharmacol. Res. Perspect..

[45] Lee W.K., Reichold M., Edemir B., Ciarimboli G., Warth R., Koepsell H., Thévenod F. (2009). Organic cation transporters OCT1, 2, and 3 mediate high-affinity transport of the mutagenic vital dye ethidium in the kidney proximal tubule. Am. J. Physiol. Ren. Physiol..

[46] Belzer M., Morales M., Jagadish B., Mash E.A., Wright S.H. (2013). Substrate-dependent ligand inhibition of the human organic cation transporter OCT2. J. Pharmacol. Exp. Ther..

[47] Minematsu T., Iwai M., Umehara K., Usui T., Kamimura H. (2010). Characterization of human organic cation transporter 1 (OCT1/SLC22A1)- and OCT2 (SLC22A2)-mediated transport of 1-(2-methoxyethyl)-2-methyl-4,9-dioxo-3-(pyrazin-2-ylmethyl)- 4,9-dihydro-1H-naphtho[2,3-d]imidazolium bromide (YM155 monobromide), a novel small molecule survivin suppressant. Drug Metab. Dispos..

[48] Barendt W.M., Wright S.H. (2002). The human organic cation transporter (hOCT2) recognizes the degree of substrate ionization. J. Biol. Chem..

[49] Chen L., Shu Y., Liang X., Chen E.C., Yee S.W., Zur A.A., Li S., Xu L., Keshari K.R., Lin M.J. (2014). OCT1 is a high-capacity thiamine transporter that regulates hepatic steatosis and is a target of metformin. Proc. Natl. Acad. Sci. USA.

[50] Chiba S., Ikawa T., Takeshita H., Kanno S., Nagai T., Takada M., Mukai T., Wempe M.F. (2013). Human organic cation transporter 2 (hOCT2): Inhibitor studies using S2-hOCT2 cells. Toxicology.

[51] Sugano K., Kansy M., Artursson P., Avdeef A., Bendels S., Di L., Ecker G.F., Faller B., Fischer H., Gerebtzoff G. (2010). Coexistence of passive and carrier-mediated processes in drug transport. Nat. Rev. Drug Discov..

[52] Sayyed K., Camillerapp C., Le Vée M., Bruyère A., Nies A.T., Abdel-Razzak Z., Fardel O. (2019). Inhibition of organic cation transporter (OCT) activities by carcinogenic heterocyclic aromatic amines. Toxicol. In Vitro.

[53] Sandoval P.J., Zorn K.M., Clark A.M., Ekins S., Wright S.H. (2018). Assessment of Substrate-Dependent Ligand Interactions at the Organic Cation Transporter OCT2 Using Six Model Substrates. Mol. Pharmacol..

[54] Kimura N., Masuda S., Katsura T., Inui K. (2009). Transport of guanidine compounds by human organic cation transporters, hOCT1 and hOCT2. Biochem. Pharmacol..

[55] Bakhiya A., Bahn A., Burckhardt G., Wolff N. (2003). Human organic anion transporter 3 (hOAT3) can operate as an exchanger and mediate secretory urate flux. Cell. Physiol. Biochem..

[56] Xiao C., Cant J.P. (2003). Glucose transporter in bovine mammary epithelial cells is an asymmetric carrier that exhibits cooperativity and trans-stimulation. Am. J. Physiol. Cell Physiol..

[57] Chien H.C., Colas C., Finke K., Springer S., Stoner L., Zur A.A., Venteicher B., Campbell J., Hall C., Flint A. (2018). Reevaluating the Substrate Specificity of the L-Type Amino Acid Transporter (LAT1). J. Med. Chem..

[58] Fardel O., Le Vee M., Jouan E., Denizot C., Parmentier Y. (2015). Nature and uses of fluorescent dyes for drug transporter studies. Expert Opin. Drug Metab. Toxicol..

[59] Cetinkaya I., Ciarimboli G., Yalçinkaya G., Mehrens T., Velic A., Hirsch J.R., Gorboulev V., Koepsell H., Schlatter E. (2003). Regulation of human organic cation transporter hOCT2 by PKA, PI3K, and calmodulin-dependent kinases. Am. J. Physiol. Ren. Physiol..

[60] Koepp T.N., Tokaj A., Nedvetsky P.I., Conchon Costa A.C., Snieder B., Schröter R., Ciarimboli G. (2021). Properties of Transport Mediated by the Human Organic Cation Transporter 2 Studied in a Polarized Three-Dimensional Epithelial Cell Culture Model. Int. J. Mol. Sci..

[61] Jouan E., Le Vee M., Denizot C., Da Violante G., Fardel O. (2014). The mitochondrial fluorescent dye rhodamine 123 is a high-affinity substrate for organic cation transporters (OCTs) 1 and 2. Fundam. Clin. Pharmacol..

[62] Bradford M.M. (1976). A rapid and sensitive method for the quantitation of microgram quantities of protein utilizing the principle of protein-dye binding. Anal. Biochem..

[63] Chedik L., Bruyere A., Le Vee M., Stieger B., Denizot C., Parmentier Y., Potin S., Fardel O. (2017). Inhibition of Human Drug Transporter Activities by the Pyrethroid Pesticides Allethrin and Tetramethrin. PLoS ONE.

[64] de Winter J.C.F. (2013). Using the Student’s *t*-test with extremely small sample sizes. Pract. Assess. Res. Eval..

