# Supplementary material for: Substrate-Dependent Trans-Stimulation of Organic Cation Transporter 2 Activity
Source: Int J Mol Sci. 2021 Nov 29;22(23):12926. doi: 10.3390/ijms222312926 (PMC8657912; doi:10.3390/ijms222312926)
Supplement: Supplementary file 1 [file ijms-22-12926-s001.zip › ijms-1466740-supplementary.pdf]

Supplementary Material

IJMS Lefevre et al

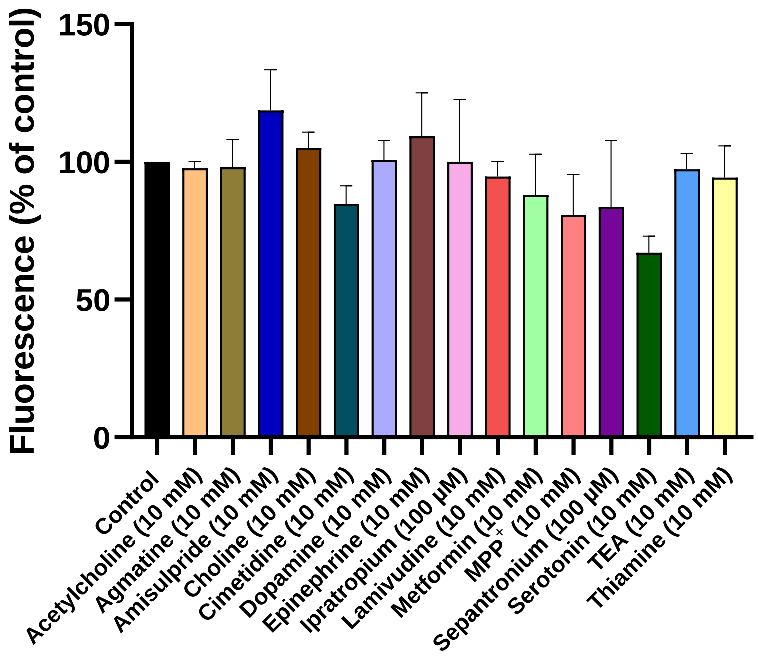

Figure S1. Effects of OCT2 substrates on DiASP-related fluorescence. Confluent HEK-OCT2 cells plated in 96-well plates were lysed in distilled water (155 µL/well). A 15 µL aliquot of 100 µM DiASP solution, without (control) or with OCT2 substrates (each used at the concentration indicated on the graph), was next added by well. DiASP-related fluorescence was finally determined by spectrofluorimetry. Data are expressed as % of fluorescence found in the absence of OCT2 substrates (control), arbitrary set at 100%, and are the means ± SEM of three assays; their statistical analysis (ANOVA followed by the Dunnett's post-hoc test) demonstrated no significant effect of OCT2 substrates towards DiASP-related fluorescence ($p>0.05$).

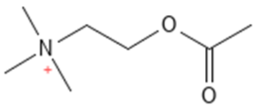

Acetylcholine

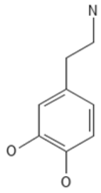

Dopamine

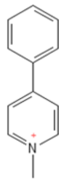

MPP⁺

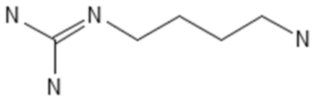

Agmatine

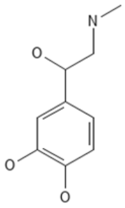

Epinephrine

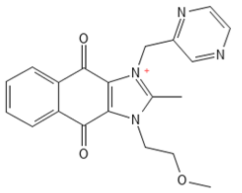

Sepantronium

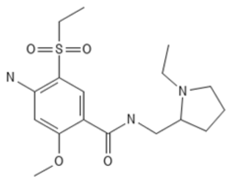

Amisulpride

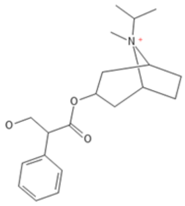

Ipratropium

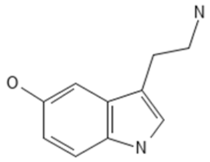

Serotonin

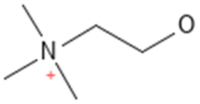

Choline

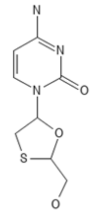

Lamivudine

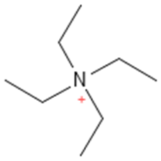

TEA

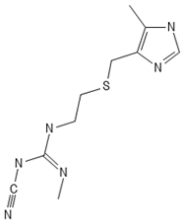

Cimetidine

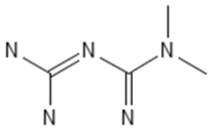

Metformin

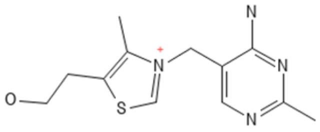

Thiamine

Figure S2. Chemical structures of OCT2 substrates

Table S1. List of analysed molecular descriptors.

Name	Description	Block
MW	molecular weight	Constitutional indices
AMW	average molecular weight	Constitutional indices
Sv	sum of atomic van der Waals volumes (scaled on Carbon atom)	Constitutional indices
Se	sum of atomic Sanderson electronegativities (scaled on Carbon atom)	Constitutional indices
Sp	sum of atomic polarizabilities (scaled on Carbon atom)	Constitutional indices
Si	sum of first ionization potentials (scaled on Carbon atom)	Constitutional indices
Mv	mean atomic van der Waals volume (scaled on Carbon atom)	Constitutional indices
Me	mean atomic Sanderson electronegativity (scaled on Carbon atom)	Constitutional indices
Mp	mean atomic polarizability (scaled on Carbon atom)	Constitutional indices
Mi	mean first ionization potential (scaled on Carbon atom)	Constitutional indices
GD	graph density	Constitutional indices
nAT	number of atoms	Constitutional indices
nSK	number of non-H atoms	Constitutional indices
nTA	number of terminal atoms	Constitutional indices
nBT	number of bonds	Constitutional indices
nBO	number of non-H bonds	Constitutional indices
nBM	number of multiple bonds	Constitutional indices
SCBO	sum of conventional bond orders (H-depleted)	Constitutional indices
RBN	number of rotatable bonds	Constitutional indices
RBF	rotatable bond fraction	Constitutional indices
nDB	number of double bonds	Constitutional indices
nTB	number of triple bonds	Constitutional indices
nAB	number of aromatic bonds	Constitutional indices
nH	number of Hydrogen atoms	Constitutional indices
nC	number of Carbon atoms	Constitutional indices
nN	number of Nitrogen atoms	Constitutional indices
nO	number of Oxygen atoms	Constitutional indices
nP	number of Phosphorous atoms	Constitutional indices
nS	number of Sulfur atoms	Constitutional indices
nF	number of Fluorine atoms	Constitutional indices
nCL	number of Chlorine atoms	Constitutional indices
nBR	number of Bromine atoms	Constitutional indices
nI	number of Iodine atoms	Constitutional indices
nB	number of Boron atoms	Constitutional indices
nHM	number of heavy atoms	Constitutional indices
nHet	number of heteroatoms	Constitutional indices
nX	number of halogen atoms	Constitutional indices
H%	percentage of H atoms	Constitutional indices
C%	percentage of C atoms	Constitutional indices
N%	percentage of N atoms	Constitutional indices
O%	percentage of O atoms	Constitutional indices
X%	percentage of halogen atoms	Constitutional indices
nCsp3	number of sp3 hybridized Carbon atoms	Constitutional indices

Table S1 (continued)

Name	Description	Block
nCsp2	number of sp2 hybridized Carbon atoms	Constitutional indices
nCsp	number of sp hybridized Carbon atoms	Constitutional indices
nStructures	number of disconnected structures	Constitutional indices
totalcharge	total charge	Constitutional indices
qpmax	maximum positive charge	Charge descriptors
qnmax	maximum negative charge	Charge descriptors
Qpos	total positive charge	Charge descriptors
Qneg	total negative charge	Charge descriptors
Qtot	total absolute charge (electronic charge index - ECI)	Charge descriptors
Qmean	mean absolute charge (charge polarization)	Charge descriptors
Q2	total squared charge	Charge descriptors
RPCG	relative positive charge	Charge descriptors
RNCG	relative negative charge	Charge descriptors
SPP	submolecular polarity parameter	Charge descriptors
TE1	topographic electronic descriptor	Charge descriptors
TE2	topographic electronic descriptor (bond resctricted)	Charge descriptors
PCWTE1	partial charge weighted topological electronic index	Charge descriptors
PCWTE2	partial charge weighted topological electronic index (bond resctricted)	Charge descriptors
LDI	local dipole index	Charge descriptors
Uc	unsaturation count	Molecular properties
Ui	unsaturation index	Molecular properties
Hy	hydrophilic factor	Molecular properties
AMR	Ghose-Crippen molar refractivity	Molecular properties
TPSA(NO)	topological polar surface area using N,O polar contributions	Molecular properties
TPSA(Tot)	topological polar surface area using N,O,S,P polar contributions	Molecular properties
MLOGP	Moriguchi octanol-water partition coeff. (logP)	Molecular properties
MLOGP2	squared Moriguchi octanol-water partition coeff. (logP^2)	Molecular properties
ALOGP	Ghose-Crippen octanol-water partition coeff. (logP)	Molecular properties
ALOGP2	squared Ghose-Crippen octanol-water partition coeff. (logP^2)	Molecular properties
SAtot	total surface area from P_VSA-like descriptors	Molecular properties
SAacc	surface area of acceptor atoms from P_VSA-like descriptors	Molecular properties
SAdon	surface area of donor atoms from P_VSA-like descriptors	Molecular properties
Vx	McGowan volume	Molecular properties
VvdwMG	van der Waals volume from McGowan volume	Molecular properties
VvdwZAZ	van der Waals volume from Zhao-Abraham-Zissimos equation	Molecular properties
PDI	packing density index	Molecular properties
BLTF96	Verhaar Fish base-line toxicity from MLOGP (mmol/l)	Molecular properties
BLTD48	Verhaar Daphnia base-line toxicity from MLOGP (mmol/l)	Molecular properties
BLTA96	Verhaar Algae base-line toxicity from MLOGP (mmol/l)	Molecular properties
